# Isolation and focal treatment of brain aneurysms using interfacial fluid trapping

**DOI:** 10.1126/sciadv.adp4579

**Published:** 2024-10-04

**Authors:** Maria Khoury, Tirosh Mekler, Mark Epshtein, Yevgeniy Kreinin, Dmitry Korneyev, Ludmila Abezgauz, Vania Anagnostakou, Guy Z. Ramon, Josué Sznitman, Matthew Gounis, Netanel Korin

**Affiliations:** ^1^ Department of Biomedical Engineering, Technion - IIT, Haifa 32000, Israel.; ^2^ Department of Radiology, New England Center for Stroke Research, University of Massachusetts Medical School, Worcester, MA 01655, USA.; ^3^ Department of Environmental, Water and Agricultural Engineering, Technion - IIT, Haifa 32000, Israel.

## Abstract

Current approaches for localized intravascular treatments rely on using solid implants, such as metallic coils for embolizing aneurysms, or on direct injection of a therapeutic agent that can disperse from the required site of action. Here, we present a fluid-based strategy for localizing intravascular therapeutics that leverages surface tension and immiscible fluid interactions, to allow confined and focal treatment at brain aneurysm sites. We first show, computationally and experimentally, that an immiscible phase can be robustly positioned at the neck of human aneurysm models to seal and isolate the aneurysm’s cavity for further treatment, including in wide-neck aneurysms. We then demonstrate localized delivery and confined treatment, by selective staining of cell nuclei within the aneurysm cavity as well as by hydrogel-based embolization in patient-specific aneurysm models. Altogether, our interfacial flow-driven strategy offers a potential approach for intravascular localized treatment of cardiovascular and other diseases.

## Teaser

Surface tension–based fluid trapping can be used to isolate and locally treat brain aneurysms.

## INTRODUCTION

Intravascular medical treatments for cardiovascular diseases are progressing to include the ability to navigate to distal anatomical locations and provide localized and targeted treatment at these sites. However, most approaches for localized treatments rely on the use of solid implants, such as stents and metallic coils for embolizing aneurysms, or on direct injection of therapeutic agents, such as an injectable embolic agent (*1*, *2*). Yet, the challenge with delivery of liquid agents, such as ethylene vinyl alcohol used to treat brain aneurysms in the past, is leakage or fragmentation distally leading to ischemic complications. The ability to provide an efficient strategy to confine the localized delivery of an injectable therapeutic to the disease site can be highly valuable for the treatment of a variety of cardiovascular diseases such as intracranial aneurysms, namely, blood-filled saccular lesions located on cerebral arteries.

Cerebral aneurysms are life-threatening lesions present in about 2 to 5% of the population, spanning 170 million people worldwide (*3*). As neuro-imaging procedures are being used more frequently, more aneurysms are being incidentally detected. Fortunately, most aneurysms remain silent and only 0.2% rupture (*4*). However, when a cerebral aneurysm ruptures, mortality is high, reaching 60% within 6 months with survivors often suffering from long-term disabilities (*4*, *5*). Current interventional treatment methods for cerebral aneurysms are mainly catheter based and include coiling and stenting (i.e., 80% of the cases) (*6*), while surgical clipping is used in the remaining cases, all of which involve potential risks of complications. In addition, some aneurysms are difficult to treat because of their irregular shape (e.g., large neck aneurysms), size, extent of aneurysm thrombosis, and anatomical location, thus resulting in a higher rate of intraoperative complications, rupture, and lower success rates (*7*). Altogether, current preventive aneurysm repair strategies carry a risk of approximately 6 to 10% for poor neurological outcome, which for most unruptured aneurysms is substantially higher than the risk of rupture (*8*). Moreover, the use of metallic-based implants may be accompanied by dual antiplatelet medication that can increase the risk for bleeding complications.

Unlike stenting, coiling, and balloon remodeling mentioned above that rely on solid devices to isolate and embolize the diseased area (*4*), the use of injectable fluidic biomaterials has high potential for aneurysm treatment as it enables complete filling of the cavity and embolization using biodegradable materials that can provide optimal outcomes (*2*, *9*–*11*). Moreover, targeted delivery and localizing therapeutic agents at the aneurysm site can also potentially open opportunities for pharmaceutical-based treatment of aneurysms that currently do not exist. However, confining the treatment to the aneurysm cavity is challenging, and using balloon-assisted procedures, where a balloon is placed and inflated on the aneurysm neck to mechanically seal the cavity, not only increases the risk for complication but does not provide an optimal seal. Furthermore, results of such procedures are highly dependent on the skills of the surgeon performing the operation (*12*–*15*).

Here, we present a fluid-based strategy for localizing intravascular therapeutics that leverages surface tension and immiscible fluid interactions to allow confined and focal treatment at brain aneurysm disease sites. The trapping and isolation of fluid compartments using immiscible phases (IMPs), such as water/oil systems, has attracted remarkable interest across various applications, spanning from treating oil spills in the ocean (*16*) to the field of microfluidics where surface tension forces can dominate (*17*, *18*). Droplet microfluidics as well as stationary arrays of trapped liquids have been developed for various applications including diagnostics, drug discovery (*19*), and material synthesis (*20*). Moreover, the use of immiscible fluids (e.g., oil-water/gas-water) allows not only the isolation of a fluid within a confined geometry but also providing localized treatment within the trapped fluid phase. As described above, surface tension can be used to capture fluids in defined geometrical structures, while the use of immiscible fluids can be applied to phase separate and allow confined focal treatment. However, such fluid confinement and immiscible fluid dynamics have so far not been thoroughly explored in the context of bio-fluids in physiological systems (*21*, *22*), such as the cardiovascular system and more specifically toward localized treatments. Here, we show, via computational simulations and in vitro experiments in brain aneurysm models, that isolation and confined treatment can be robustly achieved. In addition, we demonstrate, using surface tension isolation, successful targeted delivery of staining agents and confined fibrin hydrogel embolization in human reconstructed physiological models.

## RESULTS

### Surface tension–based isolation of aneurysms—General principle

**Fig. 1.**
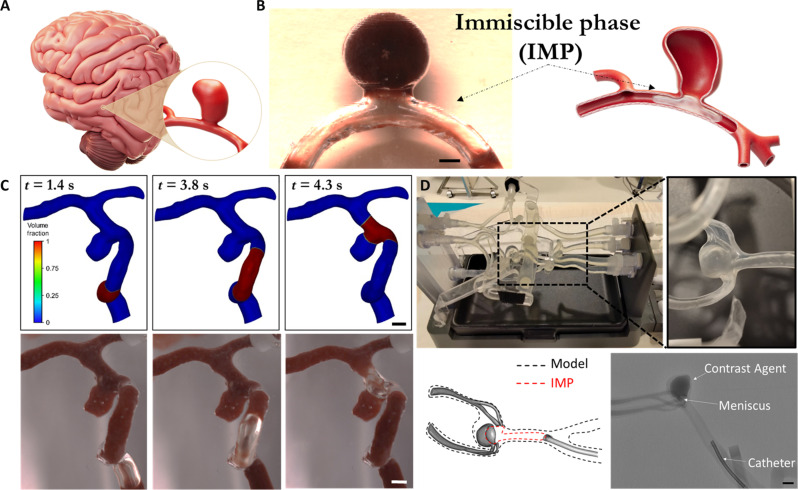
Surface tension–based isolation of aneurysm cavities. (**A**) A schematic illustration of an intracranial aneurysm. (**B**) Isolation of an aneurysm using an IMP placed under the neck of the aneurysm (left: an air IMP isolates an in vitro reconstructed aneurysm model; scale bar, 4 mm; right: illustration). (**C**) Experiments showing sequential IMP displacement inside a reconstructed patient-specific aneurysm model where the IMP is infused into the vasculature until it reaches the aneurysm site where it isolates the blood-filled aneurysm. Top: in silico results; bottom: in vitro results. Scale bar, 3 mm. (**D**) In vitro experimental system (top and bottom left) consisting of a realistic aneurysm model, reconstructed from a CT image of the parent artery, and the isolated aneurysm where an air IMP was injected through a catheter forming a meniscus and trapping contrast agent in the sealed aneurysm. Bottom left: 3D reconstruction of the contrast volume in the model showing the meniscus dome and the boundaries of the IMF. Bottom right: Digital subtraction angiography (DSA) showing lateral projection of the basilar artery with the IMF creating a meniscus and trapping contrast agent at the aneurysm sac. Scale bar, 2.5 mm.

Brain aneurysms, forming a local bulge in a weakened area in the wall of a cerebral artery, result in an unnatural blood-filled cavity (see Fig. 1A). Leveraging surface tension as the driving mechanism across this characteristic cavity structure, we propose to use an IMP positioned at the neck of the aneurysm as a method to seal and isolate the aneurysm’s cavity for further treatment (see Fig. 1B; schematic on the right and an image of an IMP, air in this case), isolating an aneurysm cavity in an in vitro model (on the left). Following the arrest of blood flow, via an upstream balloon, when an IMP is infused before the aneurysm cavity, it travels as a bulk phase confined between the vessel walls and the ambient fluid in the artery. However, as it reaches the cavity, the IMP travels across the cavity without entering the cavity, thus forming a meniscus across the aneurysm neck that is stabilized by surface tension and trapping of fluid within the cavity while isolating the aneurysm cavity. Moreover, IMP-driven aneurysm sealing can also be achieved in complex realistic configurations of brain aneurysms, as shown using computational fluid dynamics (CFD) simulations and experimental results in patient reconstructed models (see Fig. 1C and movie S1, respectively). Next, to demonstrate this method for localized isolation of aneurysms in an intravascular clinical setting, we performed experiments in a neurovascular flow system that accurately replicates human anatomies and includes a full torso and a circle of Willis comprising a realistic (berry) aneurysm, located at the basilar-p1 junction (see Fig. 1D). Fluid trapping and isolation of the berry aneurysm was achieved using an IMP (air in this case) infused to the aneurysm neck via a 5F catheter located upstream from the aneurysm. As shown in Fig. 1D, computed tomography (CT) angiograms demonstrate successful trapping and isolation of a contrast agent at the aneurysm cavity highlighting the surface tension–based isolation of aneurysms, using IMPs.

### Stability and robustness of surface tension–based isolation of aneurysm cavities

**Fig. 2.**
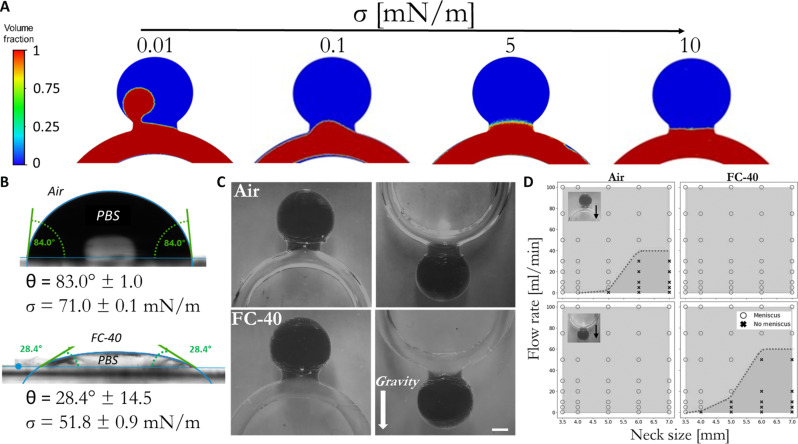
Robustness of the generated meniscus. (**A**) CFD simulations showing that the meniscus is successfully generated even at low values of surface tension (>0.1 mN/m). (**B**) Contact angle and surface tension measurements of both air and FC-40 (when PBS is the surrounding phase) using a goniometer. (**C**) Images of a successfully generated meniscus in wide-neck idealized models of aneurysms (neck = 7 mm) when IMP (air or FC-40) was injected under a flow rate of 75 ml/min, when the models were positioned either along or against gravity. Scale bar, 4 mm. (**D**) Phase graphs that summarize the conditions to generate a stable and strong meniscus for an air and FC-40 IMP as a function of the aneursym’s neck diameter, flow rate of IMP’s injection, and the aneurysm’s orientation. In all aneurysms’ sizes and configurations, a stable meniscus can be formed. Moreover, in gravity-favorable conditions even at low flow rates (0.5 ml/min), a stable meniscus forms for all the examined model geometries.

To examine the required surface tension threshold to form a stable meniscus, a typical aneurysm was modeled with an aspect ratio (AR) of 1.6, which is defined by: aneurysm neck/aneurysm height. This value was selected as previous data suggest that aneurysms with AR > 1.6 are of high risk for rupture (*23*) where almost 80% of ruptured aneurysms occur when the AR is larger than 1.6 (*24*) and thus should be treated. Accordingly, CFD simulations were performed in aneurysms with AR = 1.6 and neck = 6 mm. As shown in Fig. 2A, at very low surface (σ < 0.01 mN/m), the IMP enters with ease the aneurysm’s cavity and isolation is prohibited. However, as we increase the surface tension gradually, a meniscus is formed and stabilizes, resulting in two separated compartments. We find that at σ > 1 mN/m, a stable meniscus is formed across the aneurysm cavity, sealing and isolating the aneurysm cavity (see movie S2). Different IMP materials can provide a surface tension that notably exceeds this threshold value; see, for example, Fig. 2B showing measurements performed for a gas IMP—air [71.0 ± 0.1 mN/m against phosphate-buffered saline (PBS)] and a liquid IMP—FC-40 (51.8 ± 0.9 mN/m against PBS). To further validate the ability of IMPs, such as air and FC-40, to robustly generate a stable meniscus and isolate brain aneurysms, we performed an in vitro parametric study using aneurysm models with a spectrum of neck diameters (*N* = 3.5 to 7 mm) where the IMP was infused under a wide range of flow rates (0.5 to 100 ml/min). In addition, to account for gravitational-based forces due to density differences between the IMP and water, we examined aneurysms positioned both upward and downward. Our results show that, when air, which is less dense than water, is used as an IMP, a stable meniscus is generated when the aneurysm is in a “gravity favorable” position, that is, when the aneurysm is positioned below the parent artery. Conversely, when FC-40, which is denser than water, is used, a gravity-favorable condition is achieved when the aneurysm is positioned above the parent artery. In gravity-favorable conditions, a stable meniscus was always formed, regardless of flow rates and aneurysm sizes (see Fig. 2, C and D).

When tested in a “gravity unfavorable” condition, where the IMP tends to settle (FC-40 and aneurysm below the artery) or float (air and aneurysm above the artery) toward the aneurysm cavity, a stable meniscus is formed for all aneurysms smaller than 5 mm (*N* < 5 mm) even at low flow rates (0.5 ml/min), while for larger aneurysms with larger necks (5 < *N* < 7 mm), a higher flow rate is required (flow rate > 50 ml/min) (see Fig. 2, C and D, figs. S1 to S3, and movie S3).

Furthermore, a stable meniscus was generated in aneurysms with AR > 1.6 and different radii of curvatures as well (figs. S4 and S5). Thus, surface tension–based IMP fluid trapping and isolation of brain aneurysm can be robustly achieved.

### Localized confined treatment of aneurysms using fluid trapping and replacement

**Fig. 3.**
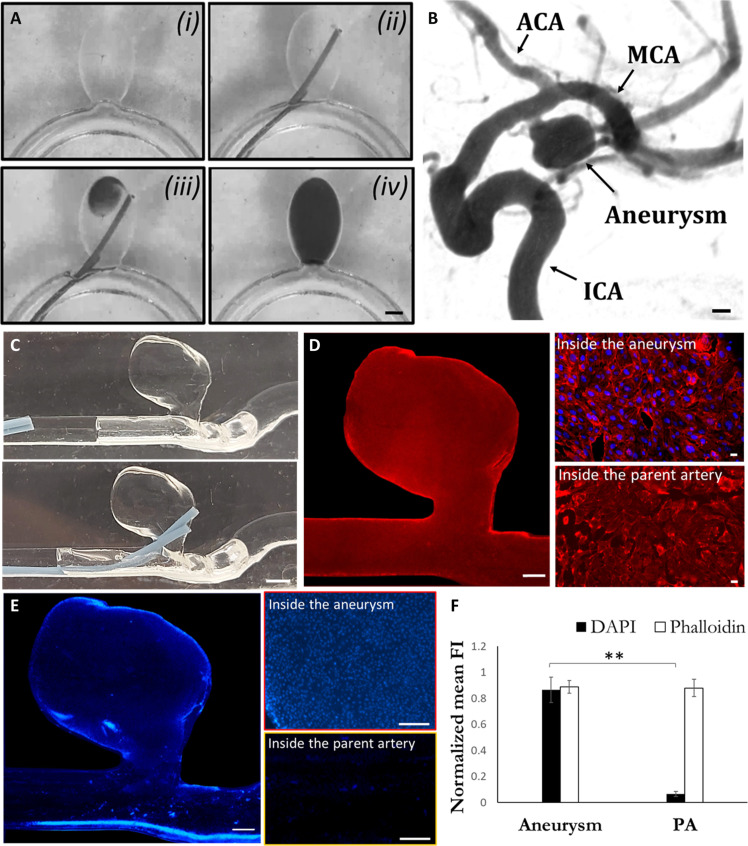
Fluid trapping and localized therapeutics in an IMP-confined aneurysm. (**A**) In vitro experiment of a direct injection and fluid replacement in an idealized aneurysm model isolated using an air IMP within 2 to 3 min. Scale bar, 3 mm. (**B**) CT image of the examined patient-specific aneurysm. Scale bar, 3 mm. (**C**) Photos of the experimental system showing the injection tubes entering (upper image) and then penetrating through the meniscus (lower picture), used for the selective delivery of the nuclei staining dye (DAPI) within the IMP confined aneurysm. Scale bar, 3 mm. (**D**) Microscope images showing actin (phalloidin) and nuclei (DAPI) staining in the endothelialized aneurysm model. Phalloidin staining was performed in the entire model before the IMP isolation and localized delivery of DAPI. Scale bar, 1 mm (left) and 10 μm (right). (**E**) Fluorescent microscopy images comparing DAPI staining of cultured endothelial inside the aneurysms and in the parent artery. Scale bar, 1 mm (left) and 200 μm (right). (**F**) Quantification of the fluorescent intensity differences in phalloidin and DAPI between inside and outside the aneurysm. Data are means ± SD (*n* = 3, three cell-cultured models under the same conditions). Significance determined using paired two-sample *t* test, **P < 0.01.

Upon isolation of the aneurysm via the IMP, it is possible to directly deliver localized injectable therapeutics across the IMP into the aneurysm while ensuring the injected material remains confined within the aneurysm. Fluid replacement within the aneurysm cavity can be performed in an isovolumetric manner by perfusing the therapeutic fluid in parallel to simultaneously withdrawing the same volume of fluid in the cavity using another tube/lumen (see Fig. 3A). Using this method, we first show the confined delivery of a water-soluble color dye, mimicking a therapeutic agent, completely filling an idealized aneurysm model confined by an air IMP to seal the aneurysm, which is performed within 2 to 3 min (see Fig. 3A). This is in contrast with the spill out observed when the same procedure was conducted without the IMP isolation (see fig. S6).

The naturally forming surface tension–guided compartmentation and its simplicity for sealing and delivering therapeutics to the aneurysm make it applicable for different types of brain aneurysm geometries and shapes and for a wide range of pharmaceutical agents and injectable biomaterials. To demonstrate localized delivery of an active chemical agent and its confinement to human brain aneurysms, we performed experiments in an endothelialized patient reconstructed aneurysm model (see Fig. 3B) showing the CT scan of the selected patient specific aneurysm [AnueriskWeb (*25*)]. Fluid replacement and trapping of nuclei staining dye, DAPI (4′,6-diamidino-2-phenylindole), within the air IMP isolated aneurysm (see Fig. 3C) resulted in confined, selective staining of the nuclei within the patient specific aneurysm cavity. As evident by fluorescent microscopy images and its quantification when compared to the parent artery showing a >10 times higher signal in the aneurysm (*P* < 0.05), the control staining of actin in the entire model showed no difference between the artery and aneurysm cavity (see Fig. 3, D to F, and fig. S7).

These results emphasize the strength of the surface tension strategy to localize treatments, which can be valuable for future pharmacological therapy within aneurysms (*26*, *27*). In addition, this multicompartment approach can be used sequentially and thus deliver several injectable biomaterials that can interact specifically with the aneurysms, such as delivering two-component hydrogel-forming solutions to selectively embolize the aneurysm.

### Aneurysm embolization using surface tension–based fluid trapping

**Fig. 4.**
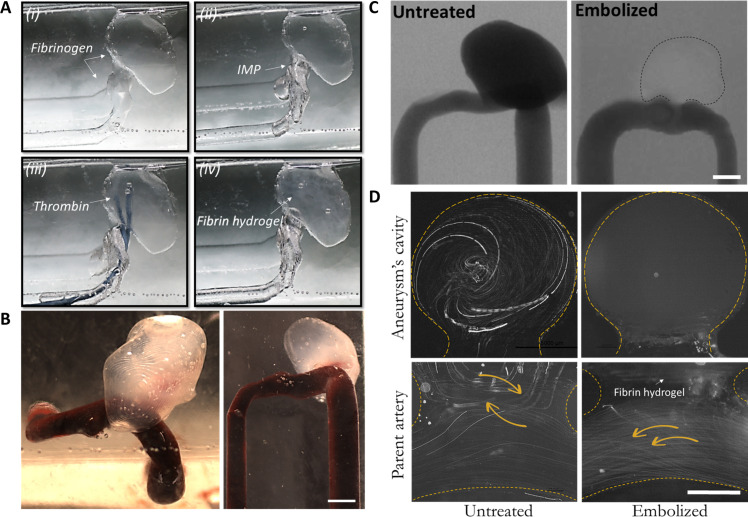
Embolization of surface tension–isolated aneurysms. (**A**) Time-lapse imaging of the fibrin hydrogel embolization procedure performed in an in vitro aneurysm model using air IMP for the isolation. Scale bar, 5 mm. (**B**) A fibrin hydrogel embolized aneurysm model following IMP isolation and treatment. Blood is perfused through the parent artery while not entering the fully embolized aneurysm (scale bar, 5 mm). (**C**) CT angiography images of untreated (left) and the fibrin embolized (right) reconstructed aneurysm model showing full embolization of the aneurysm via the surface tension isolation method where no contrast agent is observed in the cavity. Scale bar, 5 mm. (**D**) Images of pathlines of 10-μm polystyrene particles flowing under physiological flow through the parent artery entering the aneurysm in the untreated aneurysm (left) while not showing any flow into the fibrin hydrogel embolization aneurysm following IMP isolation and treatment (right). Scale bar, 2 mm.

As mentioned earlier, although the use of injectable biomaterials, such as hydrogel embolic agents, to occlude the aneurysm sac carries many advantages in repairing aneurysms, it is also complex to isolate and fill it completely while avoiding any protrusions or inadvertent leakage to the parent artery. As a proof of concept, to demonstrate surface tension–based isolation and confined embolization of aneurysms, we have used fibrinogen and thrombin solutions, where thrombin acts as a cross-linking agent to convert fibrinogen into fibrin and form a fibrin hydrogel that can successfully occlude the aneurysm cavity (see movie S4). First, the aneurysm and the parent artery are filled with a fibrinogen solution (44 mg/ml), then an air IMP is placed over the aneurysm neck to form the meniscus that isolates the aneurysm cavity and lastly thrombin (1.8 mg/ml) is introduced into the cavity over 30 s, via tubes that allow infusion withdrawal. Following the injection of the thrombin solution, within 2 to 5 min, a fibrin hydrogel is formed, and the aneurysm becomes fully embolized (Fig. 4A). To ensure efficient embolization, human blood was perfused through the parent artery, and no blood was seen to enter the cavity area (see Fig. 4B). In addition, CT angiography (CTA) images of the model (Fig. 4C), as well as three-dimensional (3D) reconstruction based on CTA, showed that the confined IMP-based sealing produced complete embolization with no protrusion to the parent artery. Eventually, the efficient embolization was further confirmed via monitoring the pathlines of flowing fluorescent particles that showed recirculating structures in the aneurysm cavity before treatment, while, following the embolization, no flow structures were observed in the cavity and pathlines were confined to the parent artery region (see Fig. 4D).

## DISCUSSION

Here, we present a strategy to nonmechanically isolate brain aneurysms without the use of implants or adjunctive devices, by positioning an immiscible fluid along the aneurysm’s neck, which allows focal and confined treatment of the aneurysm cavity. Our findings show that an IMP can robustly isolate the aneurysm cavity, including in wide-neck aneurysm (*N* > 4 mm). In addition, following the confinement, we demonstrate that a therapeutic agent can be administrated within the cavity and remain localized in the aneurysm as the IMP seals it. Moreover, we show successful confined embolization via using the IMP to confine a fibrin-based embolic hydrogel to perfectly fill the aneurysm cavity without protruding to the main vessel.

The fluid-based confinement method offers an optimal seal of the aneurysm without the need to apply mechanical forces on the vessel, such as in the case of using a balloon next to the aneurysm’s neck, which applies pressure and requires a more complicated procedure that can carry risk (*28*). Moreover, as the meniscus across the aneurysm forms a naturally defined barrier between the aneurysm cavity and the parent artery, sealing is self-controlled and not dependent on the skills of the operator of the procedure.

In our experimental setup, we have examined air and FC-40 as examples of IMPs for the generation of a robust and stable meniscus; however, other immiscible materials should be studied. It is important to note that although IMPs have been used in embolic agents (*2*), either as oil-based agents (such as lipiodol) or as solvent to carry copolymer embolic agents (e.g., in ethylene vinyl alcohol), in our approach, IMPs are used to isolate aneurysms and disease sites for focal treatment. Although air as an IMP has a high surface tension and ability to generate a highly stable meniscus, the use of air might be problematic owing to safety issues that may arise from potential downstream emboli. Previous studies have been done to examine the effect of air on the endothelium and showed that the large temporal and spatial shear stress gradients that occur on the endothelial surface may disrupt the endothelial cell membrane integrity and functionality (*29*), and therefore, further studies should be done to study the impact of the IMP on the endothelium. In addition, other alternatives should be examined such carbon dioxide (CO_2_), which is clinically used as an alternative to conventional iodinated contrast angiography (*27*). CO_2_ has the same surface tension properties as air (*30*), and although its flow in arteries may temporarily affect an endothelial cell’s function (*31*), it is still safe to use clinically, without any substantial long-term damage to the cells. Another important issue that needs to be addressed is the biocompatibility of the IMP because it will be in contact with blood components and endothelial cells along the blood vessels. In addition, developing a buoyant IMP that eliminates any gravity effect can also be helpful in further stabilizing the process of sealing even to aneurysm with very large necks (*N* > 7 mm). Independent of the IMP used, it is worth mentioning that the IMP is only used during the procedure and is collected back at the end of the procedure so that no IMP stays or circulates in the cardiovascular system. One more point to consider is that although blood flow was arrested before IMP injection as part of the isolation procedure, blood flow and pressure may still be present in the circulation, including downstream from the parent artery and past the aneurysm, which may affect the stability of the meniscus. Therefore, we also examined this condition in vitro and using CFD simulations by adding physiological flow and pressure (100 ml/min and 80 mmHg, respectively) and perfusing through a bypass inlet past the aneurysm. We then demonstrated successful isolation of the aneurysm via a stable IMP, followed by focal delivery to the isolated aneurysm while flow and pressure were still applied in the system, confirming the robustness and stability of the IMP (figs. S8 and S9 and movie S5). In addition, further studies should be performed to examine this approach in in vivo models per specific applications and on developing a clinical catheter-based delivery system to automatically perform the procedure—whether it is aneurysm embolization, gene delivery, or another application.

As a proof of principle, we embolized the aneurysm’s cavity using a fibrin hydrogel formed by the interaction of fibrinogen with thrombin. The system can be used for the localized treatment of aneurysm using a variety of injectable biomaterials and hydrogels that are studied for occlusion of aneurysms, including systems that are based on biodegradable polymers, shape-memory foams, gelation systems, and self-healing biomaterials (*9*, *32*). In addition, as gene therapy and other potent drugs that can act efficiently in aneurysm cavity will be developed (*26*, *33*, *34*), our surface tension approach should be used to deliver them to act locally in the aneurysm.

In conclusion, this work has explored a modality that can naturally and nonmechanically isolate aneurysms, using fluid confinement, to allow a localized and confined treatment of aneurysms. This approach may serve in the future to study and develop fluid-based strategies for the treatment of cerebral aneurysms and other vascular diseases, where localized treatment can be of high value.

## MATERIALS AND METHODS

### Preparation of 3D printed models

Models of intracranial aneurysms were 3D printed as previously described (*35*, *36*). In short, the aneurysm designs were reconstructed in a computer-aided design program (SolidWorks). The designs were printed using two different printers: Elegoo mars and FDM: Prusa mks3+. After printing the molds, a mixture of liquid Elastosil (RT 601, Wacker) with its curing agent (RT 602, Wacker) (mass ratio 1:10) was poured and stored at room temperature (RT) for 24 hours. Last, the models were immersed in acetone for 48 hours until full dissolvement of the plastic.

### Computational fluid dynamics

The simulations were conducted using Ansys Fluent 2023 R1, using the same CAD files used for 3D printing. A volume-of-fluid methodology modeled the flow of two IMPs, integrating surface tension. Assumptions of laminar and Newtonian fluid were implemented for all 3D simulations using an axisymmetric configuration, in consideration of the model’s symmetry plane. The inlet boundary had an injection flow rate of 10 ml/min, and a zero-pressure outlet was set at the outlet. Nonslip flow conditions were applied at the domain walls. The final simulation mesh, depicted in Fig. 1D, comprised 425K polyhedral cells with five wall inflation layers. FC-40 injection occurred at a rate of 3 ml/min till a 0.05-ml phase was formed, which was followed by PBS injection at the same rate. Simulations shown in Fig. 2A entailed water-water phases, using an analogous method; the final mesh had 578K polyhedral cells with five wall inflation layers. Contact angle and gravity effect were excluded in all simulations, and water was introduced at 10 ml/min. Postprocessing was conducted using postprocessing tools within Ansys Fluent. All simulation parameters are presented in tables S1 and S2. The patient-specific model was obtained from the Aneurisk database (C0005). The aneurysm is lateral (LAT) and located in the internal carotid artery (ICA).

For the simulation in fig. S8A, we used the same model and conditions (water-water, σ = 50 mN/m) that we used in Fig. 2A where we prevented flow from the initial inlet and additionally added a perpendicular inlet representing a bypass artery where physiological flow and pressure (100 ml/min and 80 mmHg, respectively) were introduced.

### Fluid trapping and isolation of berry aneurysm under CT angiogram

The realistic berry aneurysm isolation was performed using the SIM Agility simulator with a full torso and a circle of Willis anatomy (Mentice). The berry aneurysm was located at the basilar-p1 junction, which was accessed via the right vertebral artery. The femoral access was simulated using a 0.035″ guide wire (GLIDEWIRE TERUMO) over which an aspiration catheter (5F SOFIA TERUMO) was navigated to the center of the basilar artery through which the contrast (Omnipaque 350, GE Healthcare) and the IMP (air) were manually injected. The left vertebral artery was also blocked with a balloon catheter (accessed similarly through the left femoral artery). The injections were done manually through a 10-ml syringe connected through a stopcock to a rotating hemostasis valve. First, the vessels were filled with contrast and then IMP was slowly injected while filling the whole catheter and monitoring the presence of the IMP with fluoroscopy. The IMP could be navigated to the neck of the aneurysms and stopped by closing the stopcock. A Philips FD20 Angio-CT was used to perform a full CT scan at two IMP injection positions to produce 3D representation of the IMP.

### Contact angle and surface tension measurements

Contact angle and surface tension between different materials were measured using a Goniometer (Drop Shape Analyzer—DSA25, KRUSS). For contact angle measurements, a sessile drop technique was applied where a droplet of PBS was formed using a 0.5-mm needle (NE44, Kruss), which was placed perpendicular to an elastosil (Elastosil, Wacker)–coated slide. The volume of the droplet was increased gradually until it was released from the needle onto the slide. The angle was captured and measured using advance for drop shape analyzers software, version 1.6-03. For surface/interfacial tension measurements, a droplet was produced using a 1.83-mm needle (NE45, Kruss) and captured. The software measured the value using the Young-Laplace fitting equation. For contact angle measurements, a PBS droplet was formed in two different surrounding phases: air and FC-40. For surface tension, a droplet of PBS was placed on elastosil-coated slides in two different surrounding phases as well (air and FC-40). The slides were incubated with 1% bovine serum albumin (BSA) solution for 1 hour at 37°C before the measurements.

### Phase diagram graph

Experiments with different neck sizes (3.5, 4, 5, 6 and 7 mm, see fig. S10) and a variety of flow rates (0.5, 1, 5, 10, 20, 50, 75, and 100 ml/min) of air and FC-40 were conducted. The models were incubated with 1% BSA (Merck) at 37°C for 1 hour and then washed and filled with PBS. Afterward, the models were connected to a syringe filled with IMP, i.e., air or FC-40. The syringe was placed on a syringe pump and different perfusion rates were set. Under each flow rate in every model, the generation of the meniscus was examined. The images and videos were taken using a Sony IMX226 camera.

### In vitro experimental flow system

The experimental system included the 3D aneurysm model, three syringe pumps, two polyether-ether-ketone (PEEK) tubes (ISI), and an outlet tube (see fig. S11). The first syringe pump was used for the injection of the IMP, another for the injection of the biomaterials to the aneurysm’s cavity, and the last one was for withdrawing the fluid that was already present in the aneurysm. The two PEEK tubes were connected to two of the syringe pumps for the injection and withdrawing of the fluids from the aneurysm. Using a three-way connector, the syringe with the IMP was connected to the other two tubes and the inlet of the 3D model. The 3D model was filled with PBS upon connection to the three-way connector. The IMP was perfused to the model until it reached the aneurysm neck, forming a stable meniscus. Then, the two PEEK tubes were inserted through the meniscus, to the aneurysm’s cavity. The two syringe pumps were turned on simultaneously for the injection and withdrawing of the fluids. Once the biomaterial filled the aneurysm, the tubes were pushed back to the inlet of the model.

### Injection of IMP in aneurysm models and formation of a meniscus

The aneurysm model was filled with fluid (either blood or water) including the aneurysm’s cavity and then IMP (either air or FC-40) was injected into the model, followed by an injection of the fluid to trap the IMP between fluid phases. The injection of the fluid and the IMP was done using syringes connected to syringe pumps as mentioned above. The amount of injected IMP depends on the geometry and volume of the model and parent artery, and it should completely cover the neck of the aneurysm. For the ideal and physiological models, 300 to 500 μl of IMP was injected. For blood experiments, whole blood was taken from healthy human volunteers and supplied by the Israeli National Blood Service [Rambam Medical Center Institutional Review Board (IRB) RMB-0413-21].

### Cell culturing and seeding

Human umbilical vein endothelial cells (HUVECs; Lonza, Walkersville, MD) were cultured and maintained in endothelial cell medium (ScienCell, Carlsbad, CA), in 150-cm flasks, in a 37°C humidified environment, with 5% CO_2_. Cells were passaged every 4 to 5 days, at 85% confluency, using trypsin EDTA solution B (Biological Industries). The seeding of the HUVECs inside the 3D printed model was done as previously described (*35*, *36*). In short, the model was sterilized in UV radiation and coated with fibronectin (5 mg/ml; 1:10) for 1 hour at 37°C and then it was washed with endothelial cell medium. Afterward, HUVECs (~5 × 10^6^ cells, passage <6) were seeded inside the model, which was then placed on a rotator at 37°C for 48 hours (the number of the seeded cells depends on the size of the model used). Thereafter, the models were washed with PBS (Sigma-Aldrich) and fixed with 4% paraformaldehyde (Sigma-Aldrich).

### Cell staining inside the 3D aneurysm model

Cells were permeabilized with 0.5% Triton X-100 for 5 min at 4°C and then washed three times with PBS. Afterward, they were incubated with phalloidin (Thermo Fisher Scientific, 1 mg/ml, 1:20 in PBS) for 40 min, at RT, and then washed three times with PBS.

### IMP-based staining inside the aneurysm cavity and image analysis

The 3D cultured model, previously stained with phalloidin, was connected to the experimental system as described above. After injection of the IMP (i.e., air) and insertion of the tubes, DAPI (Thermo Fisher Scientific, 1:1000) was injected to the aneurysm cavity for 5 min in RT (using the syringe pump as described above) and then washed three times with PBS at a rate of 0.3 to 0.5 ml/min. Then, the model was placed under a microscope (Nikon ECLIPSE *Ti*) to image the model in different regions using a 0.5× objective. Images of the stained cells in the parent artery and aneurysm cavity were taken. For the fluorescence intensity measurements, different regions containing approximately 10^3^ stained cultured cells in each of the aneurysm and the parent artery were evaluated. The images were analyzed using ImageJ software, and the fluorescence intensity of phalloidin and DAPI was measured where the corrected total fluorescence was determined by subtracting out the background signal and normalizing the values (see table S3). The experiment was repeated in three cell-cultured models under the same conditions (*n* = 3).

### Formation of fibrin hydrogel using the in vitro experimental flow system

The 3D aneurysm model was filled with a fibrinogen solution (44 mg/ml) and connected to the experimental system as described above. After the injection of the IMP (i.e., air) and insertion of the two tubes to the cavity, thrombin (1.8 mg/ml) was perfused using a syringe pump at a flow rate of 300 μl/min while the other pump is withdrawing at the same rate. After 30 s of infusion, the infusion was stopped, and the tubes were taken out of the aneurysm cavity. Following this, the hydrogel was allowed to solidify over 2 to 5 min and then the air was withdrawn, and the parent artery was filled with blood while the hydrogel embolic agent remained inside the aneurysm cavity. For blood experiments, whole blood was taken from healthy human volunteers and supplied by the Israeli National Blood Service (Rambam Medical Center IRB RMB-0413-21).

### Flow pathline images

Pathlines were produced using 10-μm fluorescent carboxylated polystyrene particles (Thermo Fisher Scientific) at a concentration of 0.125 μg/ml. The elastosil aneurysm model was placed on the stage of a stereo microscope (Nikon SMZ25) and connected to the perfusion system previously described (*35*, *36*). Images were taken every 5 s for 10 min to monitor the particles’ pathlines.

### MicroCT scans

Aneurysm models (both untreated and occluded/embolized) were filled with contrast agent (Omnipaque 350, GE Healthcare) and placed inside the microCT system (Bruker), and images from 360° angles were recorded. 2D and 3D images were constructed using SkyScan1276 and CTvox software.

### Statistical analysis

All results are presented as mean and SD. Data were analyzed using paired two-sample *t* test with unequal variance, using Excel software, as noted in the figure legends. Statistical significance was considered when *P* < 0.05.
