# Supplementary material for: Isolation and focal treatment of brain aneurysms using interfacial fluid trapping
Source: Sci Adv. 2024 Oct 4;10(40):eadp4579. doi: 10.1126/sciadv.adp4579 (PMC11451524; doi:10.1126/sciadv.adp4579)
Supplement: Supplementary file 1 — Figs. S1 to S11 Tables S1 to S3 Legends for movies S1 to S5 [file sciadv.adp4579_sm.pdf]

Science Advances
AAAS

Supplementary Materials for

Isolation and focal treatment of brain aneurysms using interfacial fluid trapping

Maria Khoury *et al.*

Corresponding author: Netanel Korin, korin@technion.ac.il

Sci. Adv. **10**, eadp4579 (2024)
DOI: 10.1126/sciadv.adp4579

The PDF file includes:

Figs. S1 to S11
Tables S1 to S3
Legends for movies S1 to S5

Other Supplementary Material for this manuscript includes the following:

Movies S1 to S5

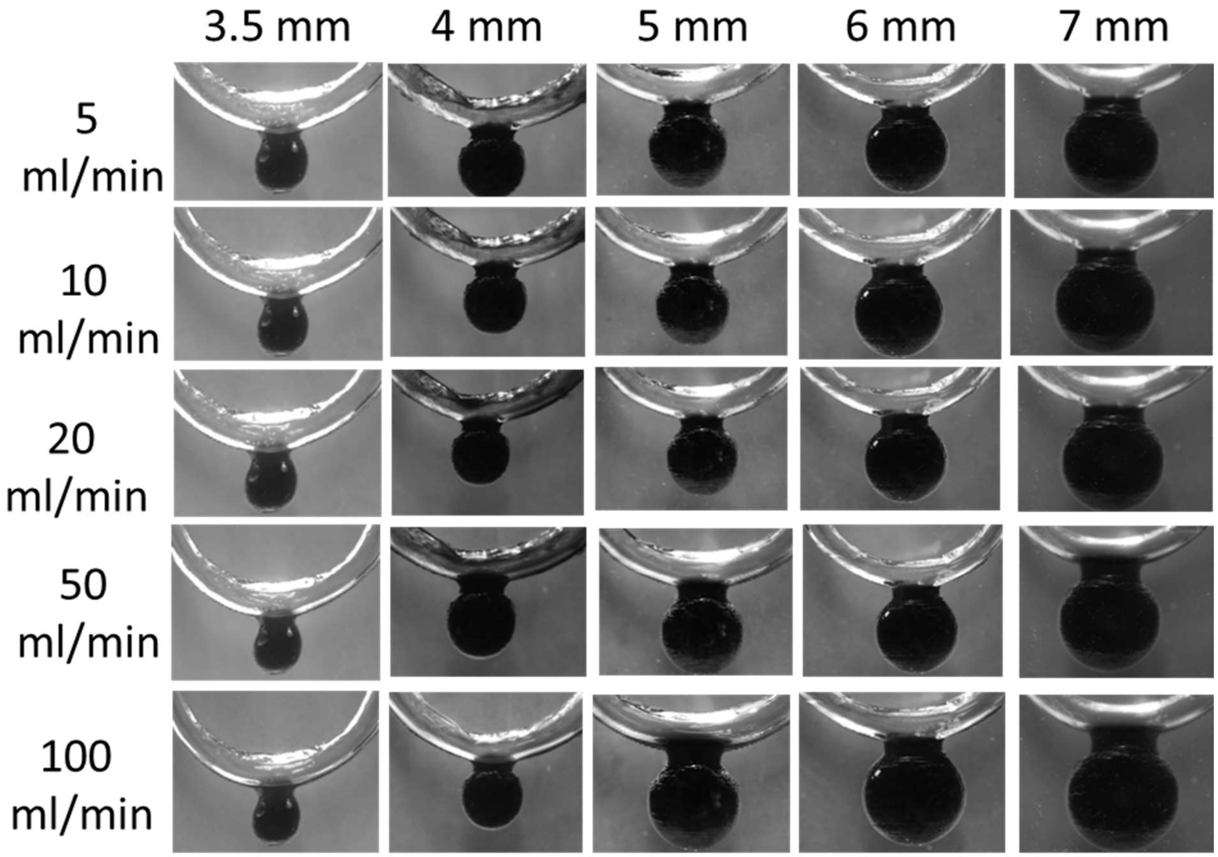

Fig. S1.

Air IMP in "gravity favorable" condition. Images of IMP aneurysm isolation in models with different neck sizes (columns) for various flow rates (rows): air IMP was injected under different flow rates in "gravity favorable" position. (Dark regions are water-soluble dye and light regions are the injected air IMP). A stable meniscus was generated under all conditions.

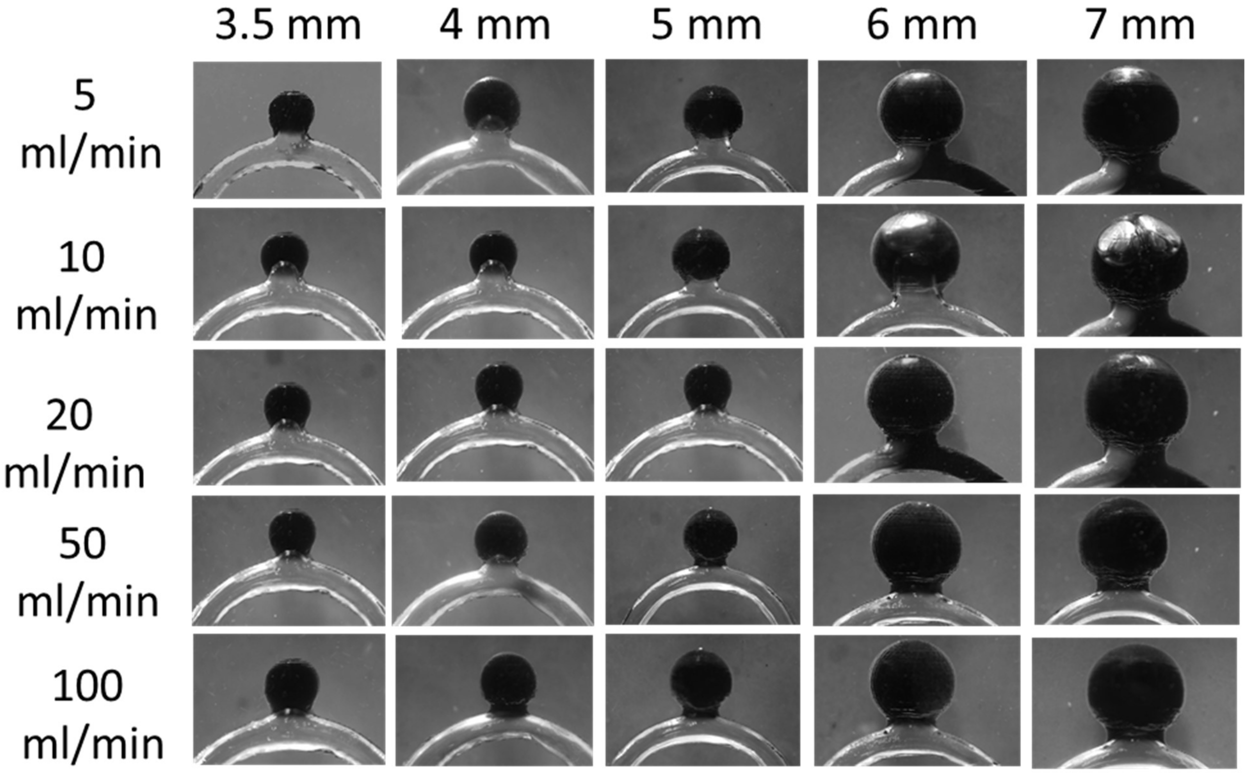

Fig. S2.
Air IMP in "gravity un-favorable" condition. Images of IMP aneurysm isolation in models with different neck sizes: air IMP was injected under different flow rates in "gravity un-favorable" position. (Dark regions are water-soluble dye and light regions are the injected air IMP). A stable meniscus is formed for all aneurysm smaller than 5 mm (N < 5 mm) even at low flow rates (0.5 ml/min), while for larger aneurysms with larger necks (5 < N < 7 mm) a higher flow rate is required (flow rate >50 ml/min).

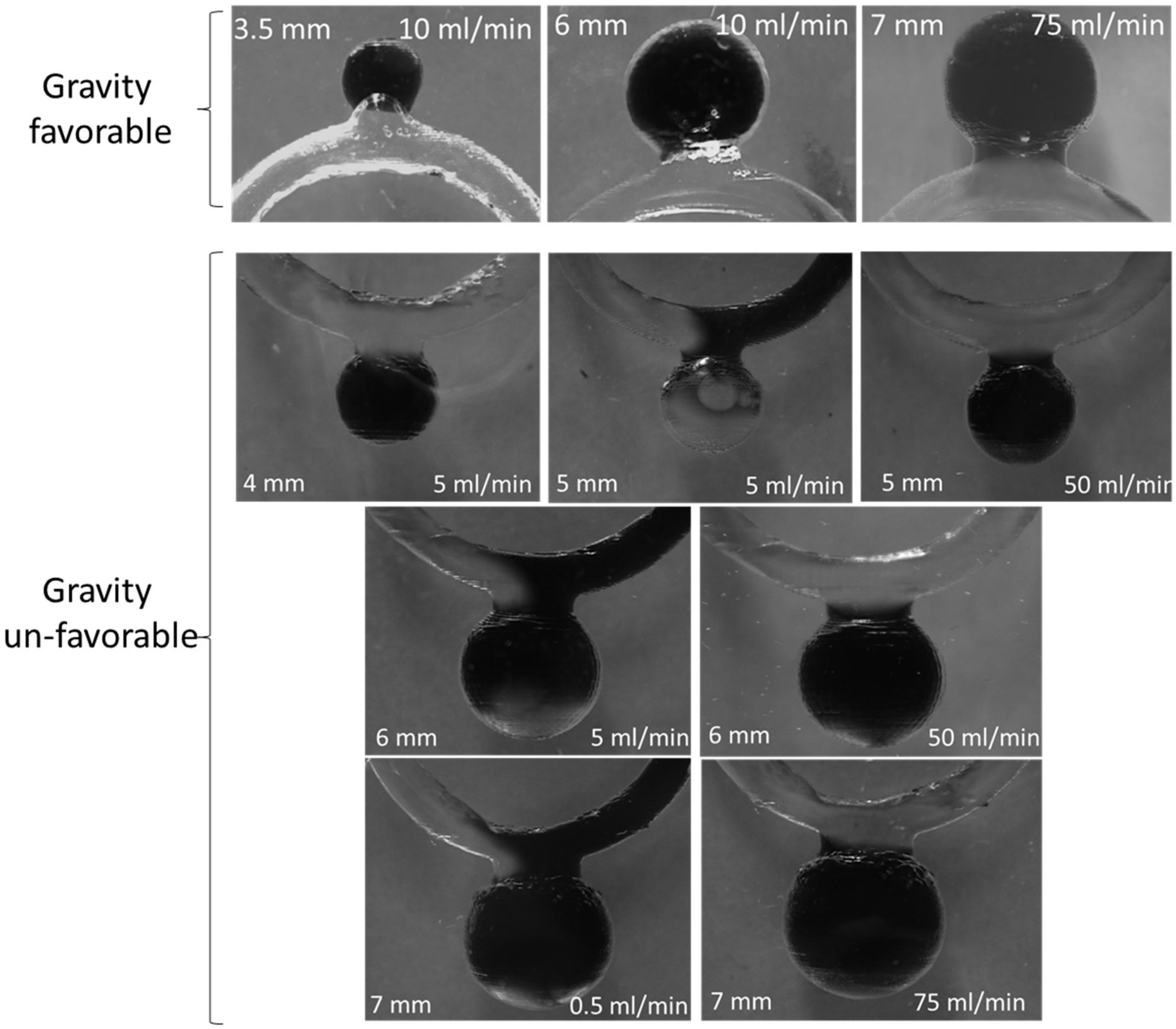

Fig. S3.

FC-40 IMP in "gravity favorable" and "gravity un-favorable" conditions. Images of IMP aneurysm isolation in models with different neck sizes: FC-40 IMP was injected under different flow rates in "gravity-favorable" and "gravity un-favorable" positions. (Dark regions are water-soluble dye and light regions are the injected FC-40 IMP). In "gravity favorable" conditions, a stable meniscus was always formed (upper panel). In "gravity un-favorable" conditions (bottom panel), A stable meniscus is formed for all aneurysm smaller than 5 mm ($N < 5$ mm) even at low flow rates (0.5 ml/min), while for larger aneurysms with larger necks ($5 < N < 7$ mm) a higher flow rate is required (flow rate >50 ml/min).

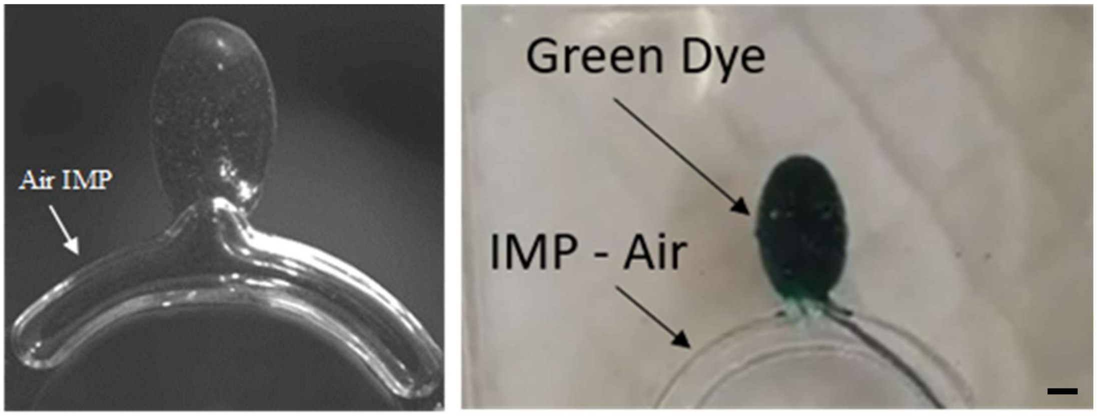

Fig. S4.
IMP isolation and focal treatment of an aneurysm with an aspect ratio of 3.2. Photo showing an aneurysm with aspect ratio of 3.2, filled with green dye after isolation with a stable air IMP generated meniscus. Scale bar: 3 mm.

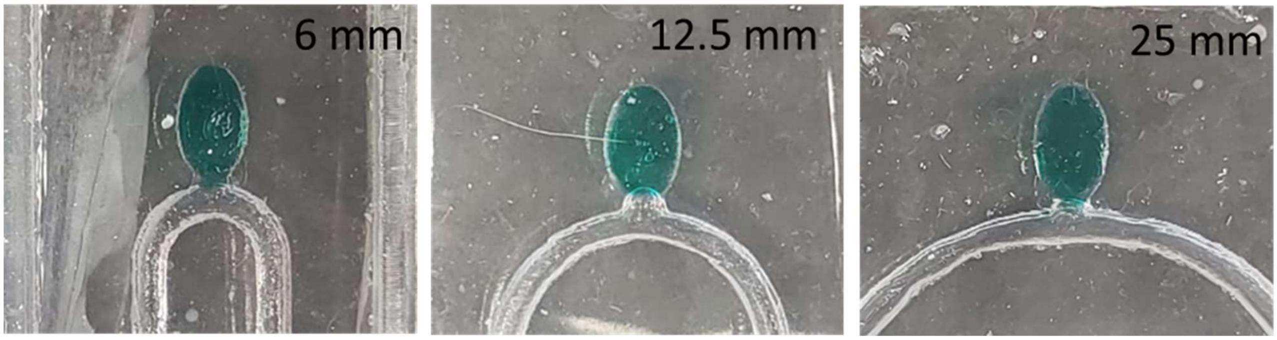

Fig. S5.
IMP isolation and confined treatment of aneurysm with parent arteries of different radius of curvature. Photos showing that meniscus was successfully generated using air as IMP in aneurysm models with different radius of curvature of the parent artery. Scale bar: 3 mm.

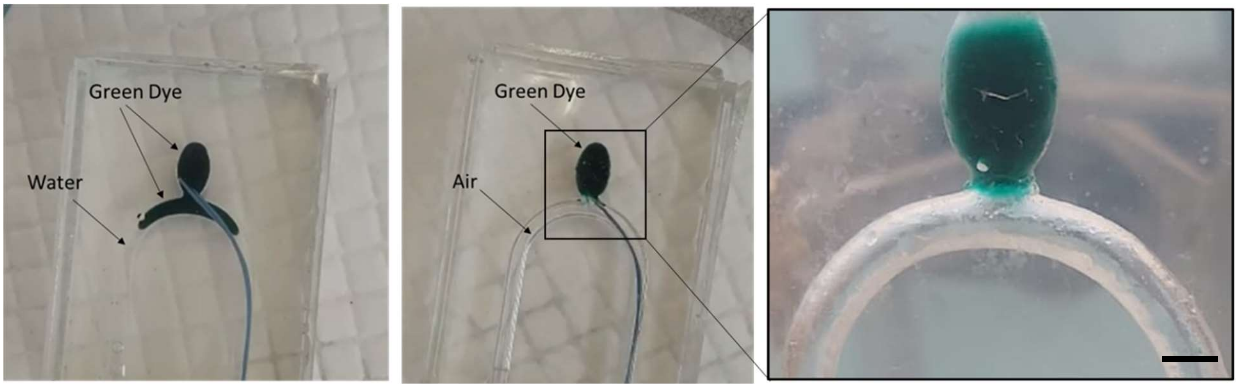

Fig. S6.

Aneurysm filling w/o meniscus. Photos showing the meniscus generated with the IMP succeeds in isolating and filling of the aneurysm's cavity (right) but leakage occurs when IMP sealing is not performed and no meniscus is present (left). The filling occurs using two tubes which are inserted into the cavity, one for the perfusion of the dye and the other withdraws at the same flow rate. Scale bar: 3 mm.

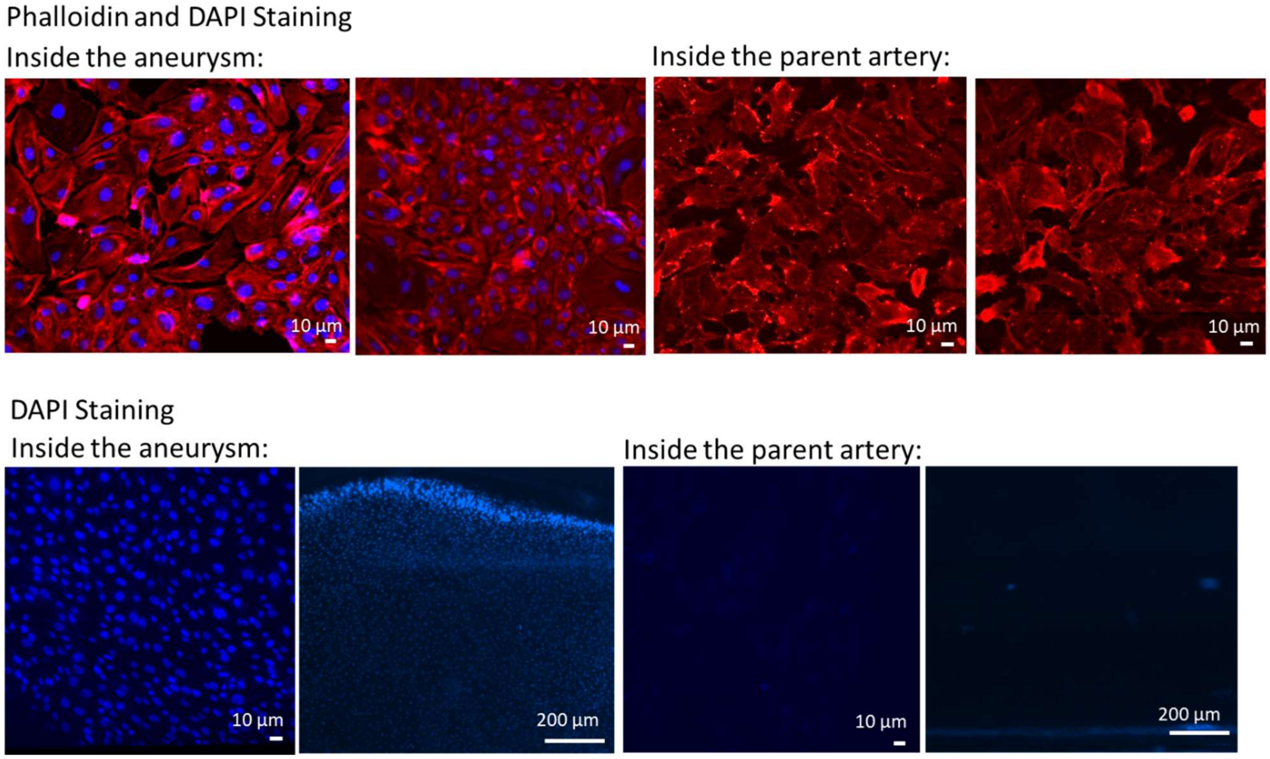

Fig. S7.
Fluorescent images inside and outside of the aneurysm following IMP confined stained for cell nuclei. Microscope images showing actin (Phalloidin) and nuclei (DAPI) staining in the endothelialized aneurysm model at different regions in each site to confirm a homogenous seeding of the cells both in the aneurysm and the parent artery.

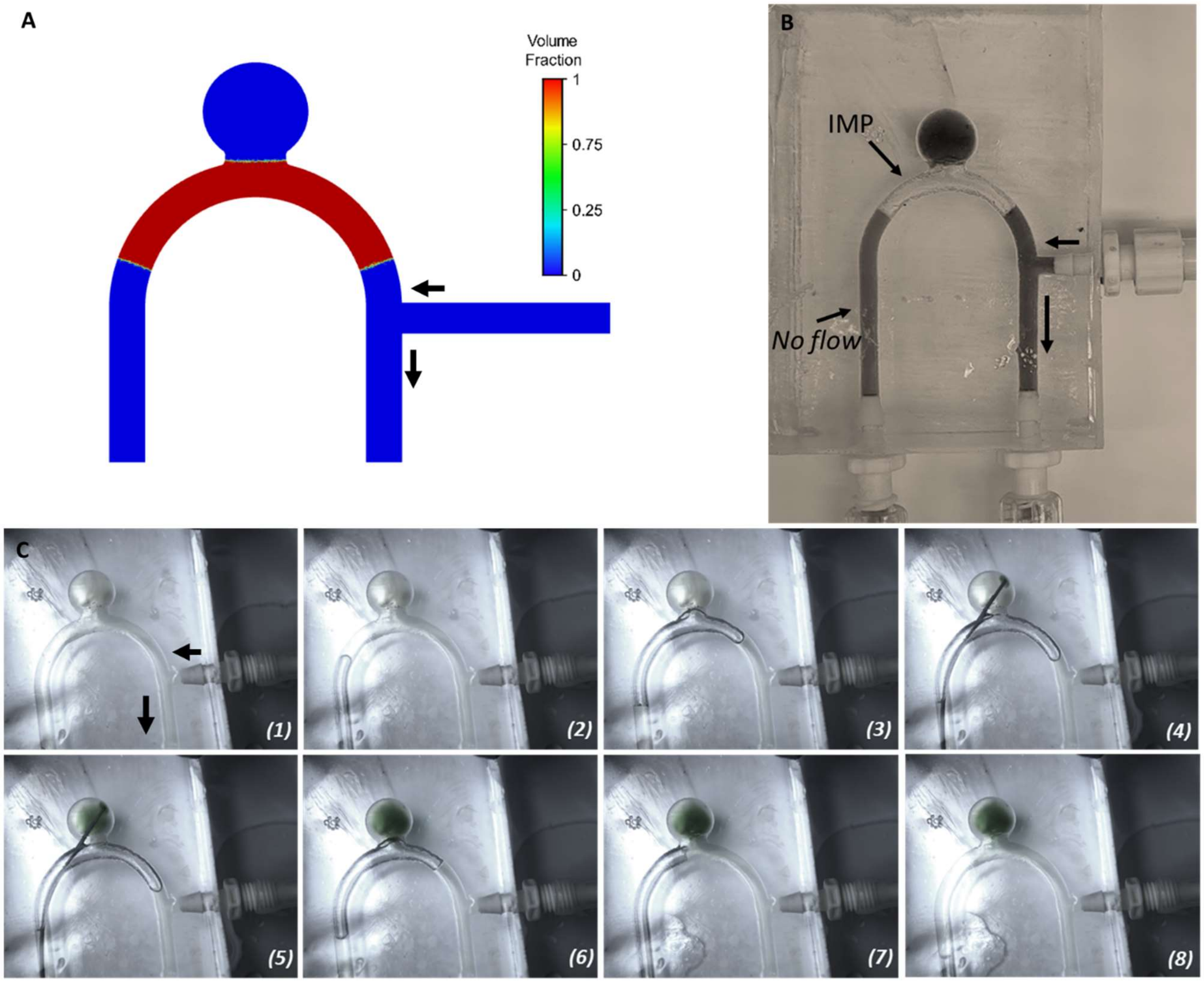

Fig. S8

Effect of physiological flow and pressure past the aneurysm site on the IMP stability and delivery to the aneurysm. (A) CFD simulation showing the stable meniscus formed over the aneurysm while flow is injected in a bypass through the inlet on the right. Arrow shows direction of flow. (B) A stable IMP was formed in an aneurysm model with an extra inlet on the right where physiological flow and pressure were applied. Arrow shows direction of flow. (C) Time-lapse images of vitro experiment showing the generation of a meniscus with a stable IMP while flow (100 ml/min) and pressure (80 mmHg) are applied from the right inlet. Bottom panel showing infusion of dye to the cavity while the aneurysm is isolated via the IMP. Scale bar: 3 mm.

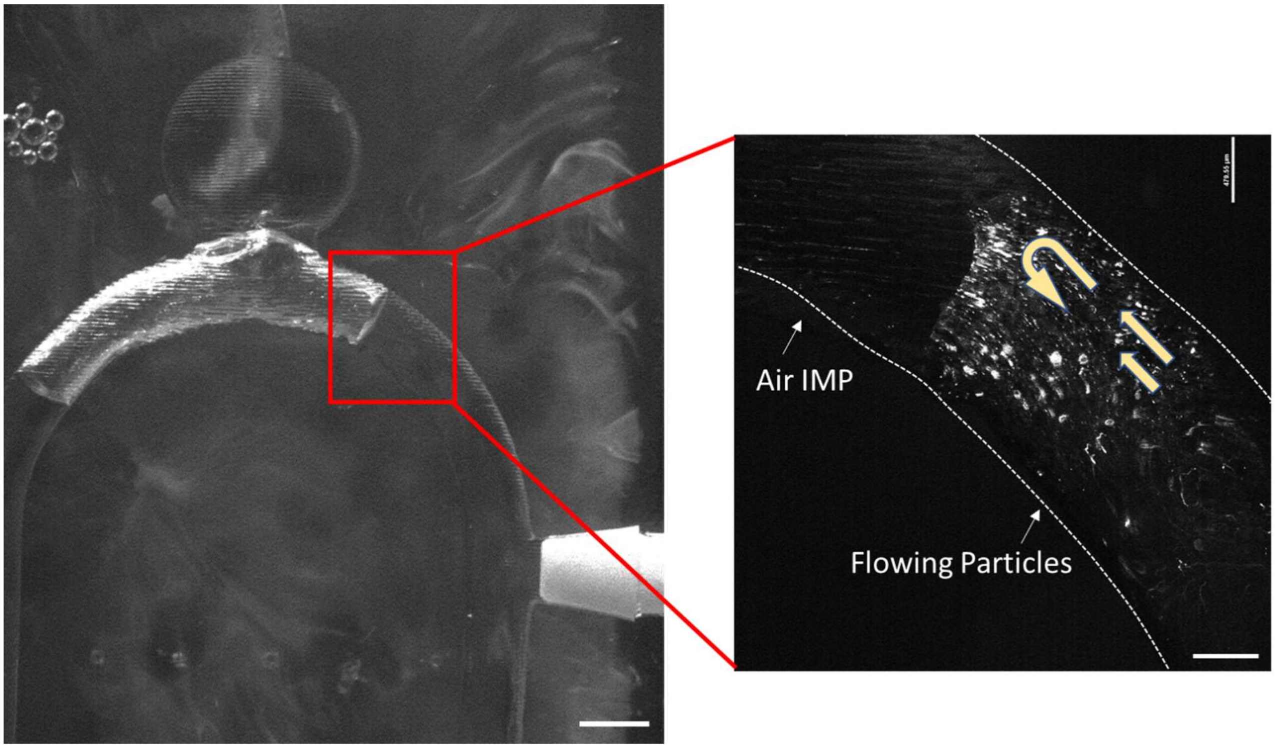

Fig. S9

A stable air IMP inside a model with flow/ pressure downstream of the parent artery. (left) a stable air IMP was injected and placed over the aneurysm neck when a solution with 2 µm particles were perfused at a rate of 100 ml/min and under a pressure of 80 mmHg, from the side inlet. (right) a zoomed-in image at the interface area between the air IMP and the particles pathlines which shows that the particles do not continue their path in the parent artery and are forced to flow downstream in the artery while the IMP remains stable. Scale bar: 3 mm (left), 500 µm (right).

	Aspect Ratio	Neck size [mm]	Height [mm]	Diameter [mm]	Radius of curvature
1	1.6	3.5	5.6	5.9	12.5
2	1.6	4	6.4	6.8	12.5
3	1.6	5	8	8.5	12.5
4	1.6	6	9.6	10.2	12.5
5	1.6	7	11.2	11.9	12.5

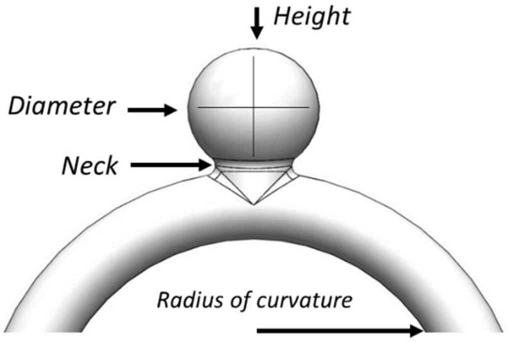

Flow Rate [ml/min]	0.5	1	5	10	20	50	75	100

Fig. S10.
Schematic and table showing the parameters for the phase diagram experiments. Different aneurysm geometries were designed to test the occurrence of a stable meniscus. Each model was subjected to a range of flow rates for the injection of the IMP.

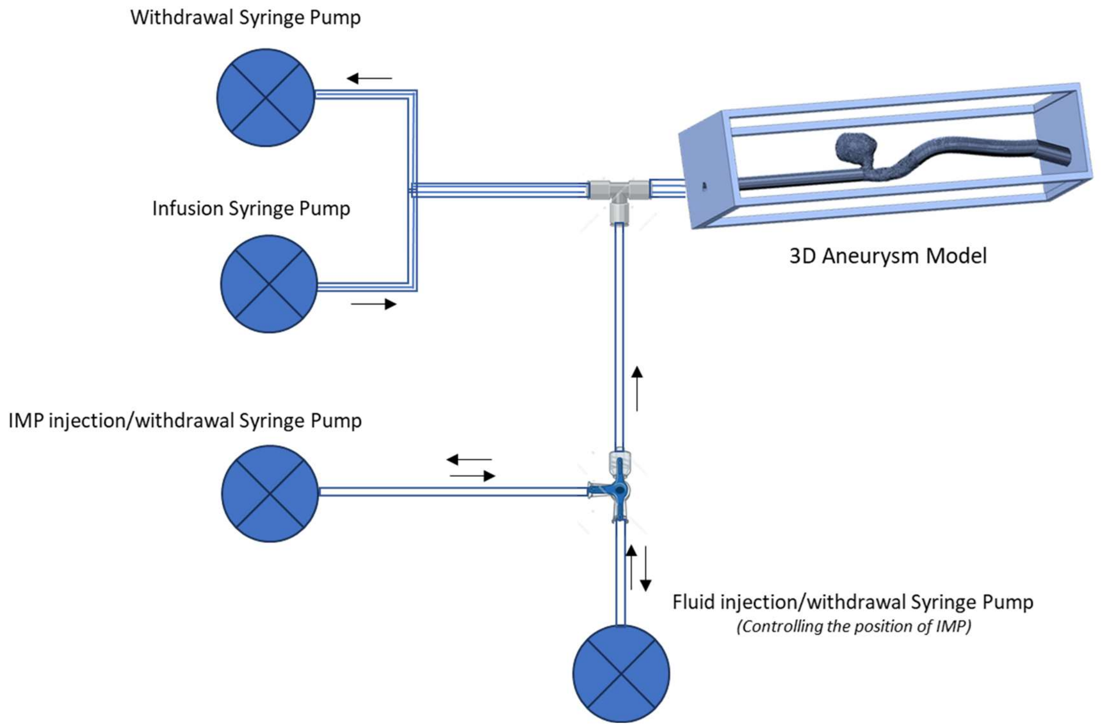

Fig. S11.
The experimental system for isolating and embolizing aneurysms. It comprises a 3D reconstructed model of the aneurysm, connected in its inlet to two tubes having syringe pumps for the injection and withdrawing of the fluids/injectable biomaterials from the aneurysm's cavity. Two additional syringe pumps are used for the injection/withdrawing of the IMP and controlling its position.

Model	Number of cells	Total volume [m^3]	Maximum volume of cell [m^3]	Minimum volume of cell [m^3]	Min. orthogonal quality
**Patient-Specific**	425K Polyhedral cells	3.15e-7	1.65e-11	4.34e-16	0.54
**Ideal**	578K Polyhedral cells	5.61e-07	1.42e-11	2.56e-15	0.25

Table S1. Simulations parameters for patient specific and ideal models.

**Patient-Specific Model**<br>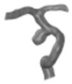			
Model: Viscous laminar inlet<br>Flow Rate: 3 ml/min<br>Outlet: zero pressure outlet<br>Wall: no slip	Material: (Water-FC-40)<br>Water:<br>Viscosity 1 cP<br>Density: 998.2 kg/m^3<br>FC-40:<br>Viscosity 4.1 cP<br>Density: 1850 kg/m^3	Multiphase: Volume of Fluid (VOF)<br>Formulation: Explicit<br>Interface Modeling: Sharp<br>Enabled:<br>Interfacial Anti-Diffusion<br>Implicit Body Force<br>Surface tension force modeling:<br>Continuum surface force (CSF)	Solver: SIMPLE<br>Gradient: Least Square Cell-Based<br>Pressure: PRESTO!<br>Momentum: Second order upwind<br>Volume Fraction: Geo-Reconstruct
**Ideal Model**<br>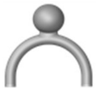			
Model: Viscous laminar inlet<br>Flow Rate: 10 ml/min<br>Outlet: zero pressure outlet<br>Wall: no slip	Material: (Water-Water)<br>Viscosity 1 cP<br>Density: 998.2 kg/m^3	Multiphase: Volume of Fluid (VOF)<br>Formulation: Explicit<br>Interface Modeling: Sharp<br>Enabled:<br>Interfacial Anti-Diffusion<br>Implicit Body Force<br>Surface tension force modeling:<br>Continuum surface stress (CSF)	Solver: SIMPLE<br>Gradient: Least Square Cell-Based<br>Pressure: PRESTO!<br>Momentum: Second order upwind<br>Volume Fraction: Geo-Reconstruct

Table S2. Parameters used in flow simulations in patient specific and ideal models.

Flourescence Intensity	DAPI		Phalloidin	
	Aneurysm	Parent Artery	Aneurysm	Parent Artery
**N=1**	0.97	0.08	0.92	0.96
**N=2**	0.84	0.07	0.92	0.85
**N=3**	0.78	0.04	0.83	0.83

Table S3. Data values of the fluorescence intensity of stained cultured HUVECSs (DAPI and Phalloidin) inside aneurysm models.

Movie S1. A Computational Fluid Dynamic (CFD) simulation showing the isolation of a physiological aneurysm model using an air IMP. An IMP is injected to the parent artery in a physiological aneurysm model. The IMP reaches the aneurysm, forming a meniscus at its neck and isolating the aneurysm from the parent artery region.

Movie S2. A Computational Fluid Dynamic (CFD) simulation showing no formation of a meniscus at very low surface tension (σ = 0.01 mN/m) while at higher surface tension (σ = 10 mN/m) a stable meniscus is formed. At very low surface tension (σ = 0.01 mN/m), the IMP enters the aneurysm's cavity and isolation does not occur. However, a stable meniscus is formed when surface tension was increased, resulting in two separated compartments (σ > 0.1 mN/m).

Movie S3. Experimental time lapse movie showing successful isolation of aneurysm using an IMP. IMP (air / FC-40) is injected to the parent artery at a high flow rate of 75 ml/min inside large neck aneurysms (7 mm), successfully generating a meniscus independent of the orientation of the aneurysm.

Movie S4. Aneurysm embolization using surface tension isolation and fibrin hydrogel formation in the cavity. A meniscus was formed upon injection of air IMP and tubes were inserted to the aneurysm's cavity. A thrombin solution was perfused to a fibrinogen filled model through one of the tubes placed in the aneurysm while withdrawing of fluid from the cavity was done simultaneously, within 2-5 min a stable fibrin hydrogel was formed that successfully occluded the aneurysm cavity.

Movie S5. Aneurysm isolation using air IMP with the presence of flow and pressure in the parent artery downstream of the aneurysm site. Air IMP was injected and a stable meniscus was formed while fluid was perfused at ap physiological flow rate of 100 ml/min to the parent artery downstream of the aneurysm through the side inlet.
